# Promoter Architecture and Promoter Engineering in *Saccharomyces cerevisiae*

**DOI:** 10.3390/metabo10080320

**Published:** 2020-08-06

**Authors:** Hongting Tang, Yanling Wu, Jiliang Deng, Nanzhu Chen, Zhaohui Zheng, Yongjun Wei, Xiaozhou Luo, Jay D. Keasling

**Affiliations:** 1 Center for Synthetic Biochemistry, Shenzhen Institutes for Advanced Technologies, Chinese Academy of Sciences, Shenzhen 518055, China; ht.tang@siat.ac.cn (H.T.); yl.wu@siat.ac.cn (Y.W.); jl.deng@siat.ac.cn (J.D.); nz.chen@siat.ac.cn (N.C.); zh.zheng@siat.ac.cn (Z.Z.); 2 School of Pharmaceutical Sciences, Key Laboratory of Advanced Drug Preparation Technologies, Ministry of Education, Zhengzhou University, Zhengzhou 450001, China; yongjunwei@zzu.edu.cn; 3 Joint BioEnergy Institute, Emeryville, CA 94608, USA; 4 Biological Systems and Engineering Division, Lawrence Berkeley National Laboratory, Berkeley, CA 94720, USA; 5 Department of Chemical and Biomolecular Engineering & Department of Bioengineering, University of California, Berkeley, CA 94720, USA; 6 Novo Nordisk Foundation Center for Biosustainability, Technical University of Denmark, 2800 Kongens Lyngby, Denmark

**Keywords:** promoter architecture, promoter engineering, synthetic promoter, synthetic biology, machine learning, *Saccharomyces cerevisiae*

## Abstract

Promoters play an essential role in the regulation of gene expression for fine-tuning genetic circuits and metabolic pathways in *Saccharomyces cerevisiae* (*S. cerevisiae*). However, native promoters in *S. cerevisiae* have several limitations which hinder their applications in metabolic engineering. These limitations include an inadequate number of well-characterized promoters, poor dynamic range, and insufficient orthogonality to endogenous regulations. Therefore, it is necessary to perform promoter engineering to create synthetic promoters with better properties. Here, we review recent advances related to promoter architecture, promoter engineering and synthetic promoter applications in *S. cerevisiae*. We also provide a perspective of future directions in this field with an emphasis on the recent advances of machine learning based promoter designs.

## 1. Introduction

*S. cerevisiae*, as a eukaryotic model organism, has been widely used as a host in synthetic biology because of its clear genetic background, rapid growth, easy cultivation and safety. Currently, yeast cell factories have been developed for the production of recombinant proteins, biofuels, natural and unnatural products, and bulk and fine chemicals [1]. The precise regulation of protein expression is important to balance the intricate metabolic pathways and to ensure a high yield of the desired products. Promoters are the basic transcriptional regulatory elements controlling the quantitative and temporal regulation of protein expression and have been extensively applied to fine-tune the expression of genes in pathway engineering in *S. cerevisiae* [2,3].

In *S. cerevisiae*, endogenous promoters can be classified into two types: constitutive promoters and inducible promoters. Constitutive promoters maintain relatively stable transcription levels with little influence from the intracellular or extracellular stimuli. In order to find strong constitutive promoters for genetic engineering, many studies have been performed to characterize endogenous promoters according to their expression strength under different growth conditions. Keren et al. [4] measured the activities of 867 native promoters which cover about one-sixth of the *S. cerevisiae* genome under different growth conditions, including various carbon sources. The promoter activities on glucose were comparable to the transcriptome data using single-molecule sequencing [5]. Commonly used constitutive promoters—including P_TEF1_, P_TDH3_, P_PGK1_, P_TPI1_, P_CCW12_, and P_ENO2_—often show slightly different strengths in various studies, which may due to an inconsistent sampling time and experimental conditions [6,7,8].

Inducible promoters can initiate a dramatic change in the transcriptional level of their corresponding genes upon the presence or absence of specific stimuli. In *S. cerevisiae*, these stimuli, or inducers, range from carbon sources, including glucose, galactose, sucrose, maltose, glycerol, acetate and ethanol, to environmental factors such as the temperature, pH, stress and light, to others, such as metabolites, amino acids, metal ions and hormones [9,10,11]. Galactose-inducible promoters, including P_GAL1_, P_GAL2_, P_GAL7_ and P_GAL10_, are considered to be strong promoters and have been used to drive a higher enzyme expression level for the efficient production of desired products [8]. By using these promoters, Ro et al. [12] constructed a heterologous pathway for the production of the antimalarial precursor artemisinic acid and Luo et al. [13] achieved the complete biosynthesis of cannabinoids and their unnatural analogues.

The construction of metabolic pathways often involves the expression of multiple genes whose expression levels span several orders of magnitude. The fine regulation of these genes makes it challenging to select the proper promoters. It is usually difficult for endogenous promoters to meet the requirements for the rational design and optimization of metabolic flux. This is mainly because the number of well-characterized promoters is inadequate, their dynamic ranges are poor, and they are often not orthogonal to endogenous regulations. For instance, P_GAL1_ is often used repeatedly for the expression of different enzymes in the construction of metabolic pathways [13], so the gene copy number has to be increased to compensate for the insufficient promoter strength [8], and the utilization of too many galactose-inducible promoters may interfere with the metabolism of galactose due to the depletion of the transcription activator Gal4p [14]. Therefore, to increase the yield of the target products and to reduce the interference from host fitness loss, it is often necessary to develop synthetic promoters for the accurate regulation of multiple genes along the foreign metabolic network. Recently, a wide range of synthetic promoters has been developed to confer constitutive, spatial or temporal gene expressions [15,16,17]. An in-depth understanding of the constitutive and regulatory mechanisms of promoters is critical for the construction of sophisticated promoters for desired gene expression patterns. In this review, we provide an introduction to the general promoter architecture and the function of its individual elements, and we summarize the design principles and metabolic engineering applications of the synthetic promoters in yeast.

## 2. Promoter Architecture and Function

**Figure 1:**
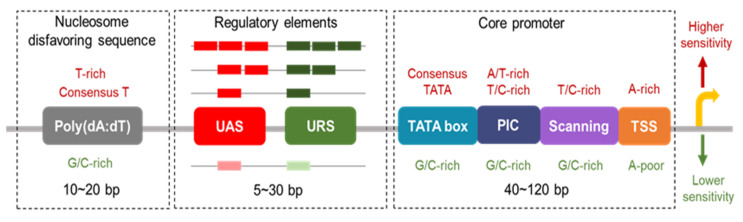
Promoter architecture in *S. cerevisiae*. The crimson rectangle represents the upstream activator sequence (UAS) with a higher activating activity and the pink rectangle is the UAS with a lower activity. Dark green represents the upstream repressor sequence (URS) with a higher repressing activity and light green represents a lower activity. The length of each element is labeled and 40–120 bp is the sequence length between the TATA box and the transcriptional start site (TSS), 5–30 bp is the UAS or URS length, and 10–20 bp is the length of the poly(dA:dT) tracts.

*S. cerevisiae* promoters have multiple essential elements for the accurate transcriptional regulation of genes, including a core promoter region, an upstream activator sequence (UAS), an upstream repressor sequence (URS) and nucleosome-disfavoring sequences, such as poly(dA:dT) sequences, as shown in Figure 1. Each of these elements plays a vital role in tuning promoter activities, thus, it is important to understand the functions and combinatorial regulatory mechanisms of these elements to predict their functions. In order to obtain a comprehensive understanding of the promoter regulatory mechanisms, several tools have been developed, such as YEASTRACT (Yeast search for transcriptional regulators and consensus tracking) and SCPD (*Saccharomyces cerevisiae* promoter database) [18,19,20,21].

### 2.1. Core Promoters

The core promoter is the nucleotide sequence that directly interacts with RNA polymerase II (pol-II) and other general transcription factors to form the pre-initiation complex (PIC) to initiate transcription. In metazoans, several conserved functional elements have been identified, including the TATA box, transcriptional start site (TSS), motif 10 element (MTE), downstream promoter element (DPE), and TFIIB recognition element (BRE), while only the TATA box and the TSS region have been identified in *S. cerevisiae*.

TATA boxes are the recognition sites of the TATA-binding protein (TBP), a general transcription factor, with a consensus sequence of TATA(A/T)A(A/T)(A/G) [22]. However, only approximately 19% of all promoters in *S. cerevisiae* contain TATA boxes. Interestingly, previous studies demonstrated that while TATA-less promoters also require TBPs for PIC assembly [23], TATA-containing promoters are highly dependent on TBP-targeted mechanisms which closely correlate to stress responses and these promoters often elicit a higher transcriptional activity [22,24,25]. TATA boxes with different sequence information affect promoter activities [26,27]. For example, Mogno et al. [24] found that the activity of a promoter containing the strong TATA box (TATATAAA) was 2.56-fold higher than the weaker one (CATTTAAA), or 4.9-fold higher than the activity of promoters without any TATA box. It was reported that the TATA box sequence TATAAA was necessary for P_HIS3_ activity and almost all single-base mutations were deleterious to its activity [28]. Besides nucleotide sequences, the location of TATA boxes is also a key determinant of its corresponding promoter activity. The activity of synthetic P_PDC1_ was higher when the TATA box docked between 88 and 66 bp upstream of the TSS, compared to between 65 and 39 bp upstream of the TSS. The TATA box was not functional when it was located 29, 19 or 9 bp upstream of the TSS, or at 19 or 9 bp downstream of the TSS [29]. Lubliner et al. [29] also found that the addition of some random flanking bases around the TATA box affected P_PDC1_ promoter activity, ranging from 24% to 132%. These results demonstrated the impacts of TATA sequences, their location and flanking bases, on the regulation of gene expression, indicating that TATA boxes can be an effective element for fine-tuning promoter activities.

The TSS region is the consensus sequence A(A_rich_)_5_NPyA(A/T)NN(A_rich_)_6_ presenting in both TATA-containing and TATA-less promoters, and the transcription is often initiated at the underlined adenosine site [30]. In *S. cerevisiae*, the location of the TSS varies from 40 bp to 120 bp downstream of the TATA box [31,32]. Between the TATA box and TSS region, a PIC region for PIC localization and a scanning region for TSS scanning by pol-II were also discovered in *S. cerevisiae* [29]. The distance variation between the TATA box and TSS mainly affects the length of the scanning region, and the PIC region is relatively constant. For example, P_GAL1_ and P_GAL10_ have different lengths between the TATA box and TSS, which are 84 bp and 114 bp, respectively, while both of their PIC regions span about 20 bp downstream of the TATA box [33]. Even though the length of the PIC region has little variation, its nucleotide sequence has a strong influence on the promoter activities. Compared to G/C-rich sequences, promoters containing A/T- or T/C-rich PIC regions possess higher activities [29]. A previous study showed that overly long scanning regions have negative effects on the promoter activity, because pol-II may need more time to search the TSS and may fall off anytime during the scanning, and so will cease the transcription initiation. The study also demonstrated that a low T content of the scanning region was negatively correlated with the promoter strength, whereas A-rich sequences overlapping with or slightly downstream of the TSS had positive effects [34].

The core promoter is one of the major determinants for the regulation of gene transcriptional levels in *S. cerevisiae*, and each of the abovementioned regions can affect the strength of its corresponding core promoter.

### 2.2. UAS and URS

The upstream activating sequence (UAS) is located upstream of the core promoter and serves as a binding site for specific transcription activators. The UAS is a crucial region of promoters which enhances gene expression. While the core promoter is responsible for PIC recruitment and assembly, the UAS provides additional stability and regulation of PIC formation [35]. All promoters recognized by pol-II may require one or more UASs for regulated gene expression [36,37]. For example, galactose-inducible promoters have various conserved UAS_GAL_ sites, a 17-bp consensus sequence 5′-CCGNNNNNNNNNNNCGG-3′ that is recognized by the transcription activator Gal4p to control their expression activities [38]. The promoters P_GAL1_, P_GAL2_ and P_GAL10_ contain four UAS_GAL_ sites, while P_GAL7_ only has one. During the induction of galactose, the production of Gal4p can improve the P_GAL1_ activity 1000-fold [37]. The upstream repressing sequence (URS) is a binding site of transcription repressors which inhibits the promoter activities. For instance, the consensus sequence 5′-SYGGGG-3′ is the recognition site of the transcription repressor Mig1p which is involved in glucose repression [39]. The activities of 5′-SYGGGG-3′ containing promoters, such as P_GAL1_, P_HXT2_, P_SUC2_, P_JEN1_, are inhibited in the presence of glucose [40,41,42,43]. Common UASs and URSs in *S. cerevisiae* are summarized in Table 1. The length of the UAS and URS vary from 5 to 30 bp and are typically 10 bp in both prokaryotic and eukaryotic organisms, including *S. cerevisiae*, because overly long sequences may have more mutational risks for their inactivation while too-short sequences may result in nonspecific genomic binding [44].

The binding affinity, quantity, and location of the UAS and URS affects promoter activities. The sequence variations of UAS and URS result in different binding affinities with their corresponding transcription factors and this has been studied in great detail using site-directed mutagenesis [79,80,81]. UAS_GAL_ is characterized by the presence of the CGG triplets at both ends, which are separated by 11 bp nucleotides. This allows for the existence of a total of 4^11^ theoretical UAS_GAL_ sites with different binding affinities. It was reported that the four UAS_GAL_ sites of *S. cerevisiae* P_GAL1_ demonstrated different activities: UAS_GAL_4 had the lowest activity, while UAS_GAL_2 and UAS_GAL_3 showed the highest activities [38]. It was reported that the affinities of UAS_GAL_ measured in an in vitro assay were inconsistent with the in vivo data [80]. Thus, the UAS_GAL_ mutant library must be characterized in vivo to understand the properties of the UAS_GAL_ sites. The saturation mutagenesis of the transcription factor Gcn4p’s binding site (5′-ATGACTCTT-3′) within the *HIS3* promoter found that almost all mismatch mutants reduced the P_HIS3_ activity significantly and only one mutant with the sequence 5′-ATGACTCAT-3′ increased the binding affinity of Gcn4p and improved the P_HIS3_ activity [82]. It has been shown that regulatory regions containing multiple UAS or URS sites for binding the same transcription factor could enhance their activation or repression of transcription. In a test of 15 transcription factors, such as Gal4p, Gcn4p, Bas1p, increasing the number of their UAS sites improved promoter activities; similarly, promoters with multiple URS sites showed a stronger repression, such as Matα2p-Mcm1p. It has also been shown that this accumulation effect will saturate in the presence of a certain number of UASs or URSs. [83,84]. It is known that UAS sites are often docked several hundred base pairs upstream of the core promoter in *S. cerevisiae* [85]. Previous studies showed that promoter activity decreased with the increasing distance of the UAS site from the core promoter in *S. cerevisiae* [83,86]. Thus, there are clear relationships between the binding affinity, quantity, location of these two regulatory sequences and the transcription level of their corresponding promoters.

The coexistence of various UASs or URSs in the same promoter could bring about the combinatorial and dynamic regulation of its transcription. Promoters of many genes related to carbon source metabolism have both URS sites, which are commonly suppressed by glucose, and UAS sites induced by other carbon sources. For example, the *GAL1* promoter contains a URS_MIG_ and four UAS_GAL_. URS_MIG_ mainly represses the activity of *GAL1* promoters under glucose growth conditions, while UAS_GAL_ induces the activity of *GAL1* promoters under galactose growth conditions. Under the condition of glucose and galactose fermentation, the regulation of *GAL1* promoters correlated to the ratio of glucose and galactose [87]. In another case, the promoter of Ime1p, a transcription factor that participates in meiosis, has a more complex regulation mechanism which is regulated by at least six URSs and four UASs for binding multiple transcription factors such as Msn1p, Msn2p, Rem1p, Sok2p, Yhp1p, and Sum1p [86]. Even though many methods have been developed for analyzing the function and interaction of UASs and URSs, the understanding of the synergistic regulation mechanisms among these different types of UAS and URS sites remains incomplete, which leads to difficulties in predicting the exact synthetic promoter phenotypes [88,89,90,91,92].

### 2.3. Nucleosomes Disfavoring Sequences at Gene Promoters

Promoters’ chromatin structure plays an essential role in transcription regulation. A low nucleosome occupancy facilitates transcription and improves mRNA abundance, while increasing the nucleosome occupancy of promoters tends to decrease their mRNA levels [93,94]. Previous studies showed that a high nucleosome occupancy can be found in numerous open reading frames and the promoters of some genes whose expression was repressed, while a low nucleosome occupancy was observed in the promoters of genes with a high expression [95,96,97]. Many studies revealed that a low nucleosome occupancy facilitates the binding of the transcription factor to the regulatory DNA sequences for the regulation of promoter activity [98,99]. For example, Gal4p interacted with its naked DNA binding sites at picomolar concentrations, while at least 100-fold more Gal4p was required to bind to the nucleosomal DNA [100,101].

Nature has evolved two main strategies to decrease nucleosome occupancy in order to regulate gene expression. One strategy involves the assistance of transcription factors, such as Rap1p, Reb1p, Abf1p and the SWI/SNF complex, which can release nucleosomes from DNA [83,101,102]. It was reported that the transcription factor Rap1p not only depleted the nucleosome from its own binding site of the *HIS4* promoter, but also reduced a nearby nucleosome to increase the accessibility of other transcription factors, including Gcn4p, Bas1p, Bas2p [102]. Another strategy is the distribution of the nucleosome-disfavoring sequences, which results in an efficient accessibility for the transcription factors [103,104]. For instance, the *PHO5* promoter has two binding sites of transcription factor Pho4p. One is a low-affinity site located in a nucleosome-free region and another is a high-affinity site occupied by a nucleosome. Under induction conditions, Pho4p interacts with the low-affinity binding site first rather than the high-affinity binding site [105,106].

The poly(dA:dT) tract, a homopolymeric stretch of deoxyadenosine nucleotides, is a well-known nucleosome-disfavoring sequence in eukaryotic organisms. Its length ranges from 10 to 20 bp, or is even greater in some cases [107]. The poly(dA:dT) tract has a low-affinity for nucleosome formation which results in nucleosome-free regions or a low nucleosome occupancy to stimulate transcription. In general, the poly(dA:dT) tract is considered an upstream activating element, not through its interaction with transcription factors, but by depleting nucleosomes [104]. Many native promoters in *S. cerevisiae* are controlled by poly(dA:dT) tracts, such as P_HIS3_, P_URA3_, P_ADH2_, P_RPS28A_ [108,109,110,111]. Modification of the poly(dA:dT) tract by changing its length, sequence information and location next to transcription factor binding sites will affect gene expression [83,112,113]. For example, P_RPS28A_ contains a poly(dA:dT) tract located 7 bp downstream of the transcription factor Abf1p binding site. Mutants of this poly(dA:dT) tract had a lower transcription of the *RPS28A* gene and the nucleosome moved closer toward the Abf1p binding site [110]. Raveh-Sadka et al. [113] has systematically analyzed the function of the poly(dA:dT) tract on promoter activities and the results showed that perfect poly(dA:dT) tracts increased transcription compared to those with two mismatches, a longer poly(dA:dT) tract with 22 bp worked better than short tracts, and an increase of the distance between the poly(dA:dT) tract and transcription factor binding site decreased the promoter activities. Thus, engineering nucleosome-disfavoring sequences, such as poly(dA:dT) tracts, may be an effective strategy for the construction of synthetic promoters with high activities to fine-tune gene expression.

## 3. Promoter Engineering Approaches

**Figure 2:**
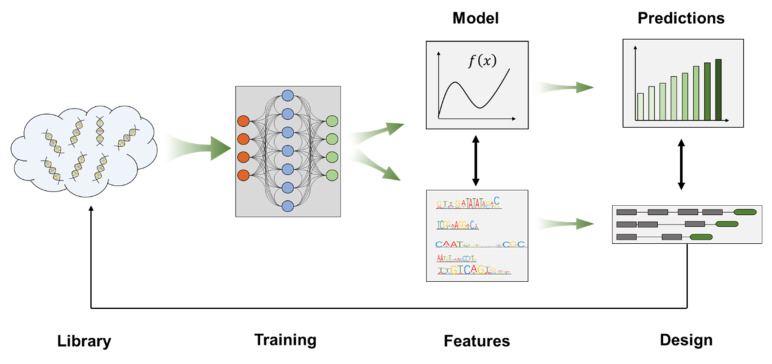
Machine learning procedures including database building, training, feature extraction (modeling), design (prediction) and verification.

Several different methods, such as a random mutagenesis by error-prone PCR, saturated mutagenesis, hybrid-promoter engineering, have been used for promoter engineering and have been reviewed previously [114,115,116]. More recently, with the introduction of machine learning into synthetic biology, great progress has been made in predicting the structure, function and interactions of biological macromolecules such as nucleic acids and proteins, and the procedure is shown in Figure 2 [117,118,119,120]. Based on big data of promoter–protein interactions, machine learning provides a new strategy for rational design and increases the predictability of promoter engineering.

Currently, machine learning is mainly used for promoter engineering in *Escherichia coli*. De Mey et al. [121] applied a partial least squares (PLS) regression method to analyze the relationship between promoter sequences and strength in *E. coli*, demonstrating the prospects of predictive and rational promoter designs. However, the accuracy for the prediction still needs improvement. Artificial neural network (ANN) models can better represent the complex and nonlinear interactions within promoter sequences and have been successfully applied in the rational design of promoters. A series of promoters with different strengths was obtained by randomly mutating the Trc promoter and its ribosome binding site in *E. coli*, which were then used for training and testing using the ANN mathematical model. Sixteen novel artificial elements were generated in silico and their predicted expression levels showed a good correlation with the experimental results, indicating that the model could be used for synthetic promoter designs with specific properties [122]. The generative adversarial network (GAN) and convolutional neural network (CNN) were also used to generate artificial promoters *de novo* and predict their expression levels, and three highly active synthetic promoters were identified in *E. coli*, among the predicted strong promoters, by experimental validation [123]. In *S. cerevisiae*, promoter libraries were created based on native promoters, including the constitutive P_TDH3_ and the inducible P_ZEV_, and a reliable prediction model was trained with the CNN based on the promoter sequence-activity data collected from these libraries, and the activity of a synthetic promoter (predicted from the *TDH3* promoter) increased by 37% and the activity of a *ZEV* promoter mutant also increased by *β*-estradiol induction, and its basal expression was reduced [124]. However, at present, few studies have applied machine learning in promoter engineering in *S. cerevisiae*.

Libraries created by random or saturated mutagenesis are dependent on the transformation efficiency of the strain. The maximal transformation efficiency of *S. cerevisiae* is about 10^8^, therefore the library capacity will not exceed the transformation efficiency. Hybrid-promoter engineering requires a lot of testing, and it is difficult to achieve a high-throughput selection due to the heavy construction workload. Machine learning could construct quantitative models based on a limited database to analyze the data distribution characteristics of the designed promoter library, helping us better understand the underlying interaction principle. The result of machine learning is to predict a serial of promoters that function as expected. This predictability can reduce the workload and increase the accuracy of testing. Therefore, it is foreseeable that machine learning methods will gain momentum in the near future and fuel the development of accurate and customer-tailored engineered promoters.

## 4. Promoter Engineering for Diverse Synthetic Promoters and Their Applications

### 4.1. Synthetic Promoters for Expanding Dynamic Ranges

**Figure 3:**
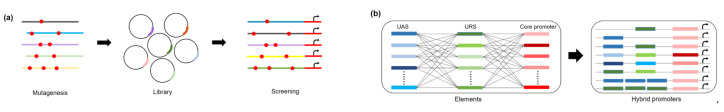
Promoter engineering for the development of synthetic promoters with dynamic ranges in *S. cerevisiae*. (**a**) Engineering promoter by random mutagenesis; (**b**) Combination of each element for hybrid promoters.

Multiple enzymes are often introduced and overexpressed in metabolic engineering to boost the yield of the desired product. However, an unbalanced expression of these enzymes would accumulate intermediates and result in unnecessary metabolic burdens or toxicities [125]. Therefore, it is necessary to precisely control enzymatic activities with the help of promoters with a wide dynamic range to ensure a balanced flux for pathway optimization. [126]. However, this field is often limited in techniques to construct a promoter set with a wide dynamic range. Currently, many studies have been focused on endogenous promoter engineering to expand the yeast promoter library to overcome these limitations. A random mutagenesis library of existing promoters has proven to be an effective method for the construction of synthetic promoters (Figure 3a). Alper et al. [15] created a library based on the *TEF1* promoter and obtained a series of synthetic promoters with a wide range of activities; the best candidate showed a two-fold higher activity than the native P_TEF1_. These promoters were used to regulate efficient glycerol production by driving the rate-limiting enzyme expression in *S. cerevisiae* [127]. Other native promoters such as P_ENO2_ and P_PDC2_ were also engineered by random mutagenesis. Synthetic P_ENO2_ and synthetic P_PDC2_ obtained from their corresponding mutagenesis libraries improved the expression of recombinant proteins cellobiose transporter and β-glucosidase for cellobiose degradation by 24.4-fold and 3.0-fold, respectively. The recombinant strain had a higher cellobiose consumption and ethanol production than its parent strain, reported as 6.41-fold and 6.36-fold more, respectively [128].

The rational combination of different promoter elements is another efficient method to improve the dynamic range of promoters (Figure 3b). Blazeck et al. [16] created a synthetic hybrid promoter by combining the strong *TDH3* promoter with three UAS_CLB_s, the 240-bp UAS sequence of the mitotic cyclin (CLB2), and this hybrid promoter enhanced the transcription level 2.5-fold compared to *TDH3* promoter. In addition, they built a series of heterozygous galactose-inducible promoters by fusing UAS_GAL_ with different core promoters and the resulting synthetic promoters had a continuous strength gradient which could achieve a fine-tuned gene expression. A series of post-diauxic phase inducible synthetic promoters were also constructed by combining core promoters and UASs, and their activities were further improved by the optimization of the UAS number [129]. More recently, engineering the nucleosome architecture of promoters has received increasing attention. Synthetic promoters with different strengths were produced by altering the properties of the poly(dA:dT) tract, including making changes to its length, composition and distance from the UAS [113]. The introduction of nucleosome-disfavoring sequences into promoters, such as P_CYC1_, P_HIS5_, P_HXT7_, and P_TEF1_, increased the strength of the resulting synthetic promoters. However, this method did not work on strong promoters such as P_TDH3_ and P_GAL1_, which may have been evolutionarily optimized for their nucleosome architecture in nature [17]. Based on such designs, synthetic promoters could acquire some expected properties such as a high activity and inducibility.

### 4.2. Synthetic Promoters for Reducing Homologous Recombination

It is well-known that *S. cerevisiae* has a strong homologous DNA recombinant capacity, which has been widely utilized in genome insertion, deletion and replacement. Multiple uses of the same promoters or their elements are prone to generating homologous recombinations and lead to the instability of synthetic pathways in *S. cerevisiae*. Thus, the development of synthetic promoters with sequence orthogonality to avoid homologous recombination is an attractive research field. Exploiting the naturally evolved diversity of heterologous promoters in *S. cerevisiae* is an effective strategy. Peng et al. [130] compared 11 galactose-inducible promoters from *Saccharomyces* species and found that most of them are stronger than *Sc*P_GAL1_, especially *Se*P_GAL2_ and *Sk*P_GAL2_ from *Saccharomyces eubayanus* and *Saccharomyces kudriavzevii*, respectively. Recently, the design of artificial promoters without the utilization of a native promoter as a scaffold has been exploited. Based on a computational approach, Curran et al. [17] predicted active promoter sequences and created six artificial promoters with a 20-fold dynamic range of transcription. The activities of these synthetic promoters were comparable to native promoters P_CYC1_ and P_HXT7_. These promoters, although still small in number, will be useful for synthetic biology applications, especially for industrial applications which are more dependent on stable gene expression.

### 4.3. Synthetic Promoters with Minimal Size

**Figure 4:**
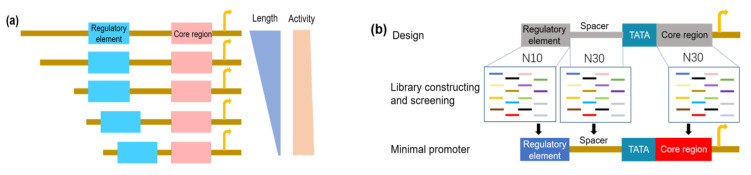
Methods for minimal promoter construction. (**a**) Rational truncation of endogenous promoters; (**b**) Artificial construction *de novo* by constructing and screening the promoter library. N30 and N10 mean the random mutation libraries of 30 and 10 consensus nucleotides, respectively.

In bacteria, such as *E. coli*, the lengths of promoters are typically less than 100 bp, whereas native yeast promoters usually span hundreds of nucleotides. The long nucleotide sequences not only decrease the efficiency of biosynthetic pathway construction, but also complicate the regulation of these pathways. The construction of minimal promoters could overcome these limitations. The truncation of endogenous promoters to remove non-essential bases is one strategy for minimal promoter construction (Figure 4a) [131,132]. *S. cerevisiae* P_TEF1_, a strong promoter commonly used in recombinant expression, was used as a model for the study of minimal promoter constructions. The results indicated that a 69 bp essential sequence can sustain detectable transcriptional activities. A series of short synthetic promoters were developed through a combination of a UAS and this short essential sequence, which achieved an 80% activity of P_TEF1_ [132]. However, the minimal promoters produced by this method also contain elements from endogenous promoters and suffer from the risk of homologous recombination. Thus, saturation mutagenesis may be a preferable method for creating minimal promoters (Figure 4b) [133,134]. In *S. cerevisiae*, to achieve minimal core promoters, sequences with different sizes (20 bp, 25 bp and 30 bp) between the TATA box and TSS were selected for saturation mutagenesis, and it was found that only the N30 library generated partially active promoters. Thirteen (13) of these functional core promoters were isolated. Furthermore, minimal constitutive UASs of 10 bp were also selected by a similar process. By combining the minimal core sequences and minimal constitutive UASs, minimal promoters were created and the activity of one of them reached 70% of the strong P_TDH3_, with approximately 20% of its original length. In addition, minimal galactose-inducible promoters were also developed by using these minimal core promoters and their activities were comparable to wild type P_GAL1_ [135]. Synthetic promoters with minimal sizes work well in *S. cerevisiae* and may have strong potential in large-scale synthetic biology applications.

### 4.4. Synthetic Promoters for Multi-Host Application

Commonly used microbial hosts in synthetic biology, such as prokaryotic *E. coli* and *Bacillus subtilis* and eukaryotic *S. cerevisiae* and *Pichia pastoris*, often have certain genetic features which make them suitable hosts for specific genetic circuits and biosynthetic pathways. However, the characterization of these circuits and pathways in different hosts always needs promoter substitution. Thus, the creation of broad-spectrum synthetic promoters for applications in diverse hosts could expand the synthetic biology toolbox to avoid promoter redesign and facilitate host selection [136]. The construction of synthetic, broad-spectrum promoters requires inter-species combinations of all basic and efficient promoter elements to enable the transcription of downstream genes in different hosts. For example, the strong synthetic minimal promoter of *S. cerevisiae* and the conserved −35 and −10 boxes from *E. coli* and *B. subtilis* were rationally combined to develop P_bs_, which could be used to drive gene expression in all three hosts. The activity of P_bs_ was much stronger than the *E. coli* strong promoter P_J23119_, comparable to the *S. cerevisiae* minimal promoter P_min_, and approximately 75% of the *B. subtilis* strong promoter P_cdd_. UAS mutations renders this promoter with an activity gradient, which is useful in biosynthetic pathway optimization [137]. Based on the information of the nucleotide distribution of *S. cerevisiae* core promoters, Portela et al. [138] successfully constructed a series of universal core promoters utilizing computational designs and library selection, and these core promoters could be used in different yeast species, including *S. cerevisiae* and *P. pastoris*. The reported broad-spectrum promoters have been used in several hosts, and more promoters of this type with a dynamic activity range are required to simultaneously drive gene expression in more diverse hosts, which would facilitate the examination of synthetic pathways in different hosts.

### 4.5. Synthetic Promoters for Constructing Biosensors

To improve the robustness of biological pathways, it is essential to design a feedback control network for gene expression regulated by metabolic intermediates or other stress factors. Gene circuits with feedback regulation usually have one or more biosensors which can respond to physical or chemical signals to realize the real-time dynamic transcription regulation, and thus automatically adjust the state of gene expression. Two key components of biosensors are transcription factors, which can detect either intracellular or environmental signals, and promoters, which can receive signals from effectors and generate an output. Thus, promoter engineering is a common method in biosensor development and optimization.

Most native promoters have some defects in terms of being a part of an excellent biosensor, therefore, engineering native promoters could increase the sensitivity and the overall performance of biosensors. Cytosolic NADPH/NADP^+^ ratios are important to maintain redox homeostasis and cell fitness. The activity of the native *TRX2* promoter, which is regulated by the transcription factor Yap1p, can be altered by sensing NADPH/NADP^+^ ratios, but its sensitivity is low. Increasing the number of the UAS-containing Yap1p binding sites greatly enhanced the cascade response effect, and this novel biosensor was useful at selecting cell populations with higher NADPH/NADP^+^ ratios [139]. Similarly, engineering the *YGP1* and *CCW14* promoters by optimizing the core promoter and the number and type of UASs successfully created a set of strong synthetic promoters for sensing low pH; these low-pH sensing promoters improved the production of lactic acid by 10-fold under low-pH fermentation compared to the native *TEF1* promoter [140]. In addition, engineering transcription factors could also change the promoter performance. In the galactose regulatory network, Gal3p responds to galactose and activates the transcription activator Gal4p to induce the transcription of galactose-inducible promoters. Gopinarayanan et al. [141] found a Gal3p mutant that could sense xylose and then regulate all galactose-inducible promoters under xylose growth conditions, allowing a better utilization and growth when using xylose as the sole carbon source.

The utilization of heterologous regulatory elements can increase the number and types of biosensors which do not interfere with the native cellular regulation of *S. cerevisiae*. The bacterial tetracycline operator (*tetO*), a DNA binding sequence of tetracycline-inducible repressors (*tetR*), has been used in numerous biosensor applications. Hybrid promoters created by the fusing of one or more *tetO* to a yeast native promoter, such as a *CYC1* promoter, were controllable under different tetracycline concentrations, and the overexpression levels of β-galactosidase comparable to P_GAL1_ are reached [142]. The number and location of the heterologous regulatory DNA sequence affect the induction ratio and the dose-response curve of biosensors, and thus their engineering is key for sensitive and robust biosensor construction. An analysis of the *tetO* locations between the TATA box and TSS found that the closer the *tetO* was located to the TATA box, the stronger the transcriptional repression; the location and number of the *tetO* together determined the dose-response curve [143]. Camphor is an inexpensive small molecule that binds to the repressor CamR, a distant homolog of the Tet repressor, to inhibit gene expression. An URS site for binding CamR was embedded within the core promoter of P_CYC1_, and a camphor-off switch was successfully constructed, which activated gene expression without camphor and repressed gene expression through the addition of micromolar concentrations of camphor, and this sensor was applied to complement the adenine-auxotrophy in a camphor-dependent manner [144]. The bacterial FapR transcriptional repressors and their cognate *fapO* DNA binding sites have also been widely used to develop various biosensors for the detection of many metabolites in *S. cerevisiae*, such as fatty acid intermediates, such as malonyl-CoA [145,146]. By combining malonyl-CoA sensors with a genome-wide overexpression library, the titer of 3-hydroxypropionic acid produced from malonyl-CoA was enhanced by 120% [146]. The successful application of heterologous elements into promoters endowed the biosensor with new properties to respond to more metabolites or chemicals, which makes it able to be used for the accurate regulation of gene expression in biosynthetic pathways.

The rational combination of different regulatory elements enables signal integration to perform the combinatorial effects of biosensors. For instance, five binding sites of the androgen receptor, which respond to steroid hormones, were placed upstream of the TATA box of the *CYC1* promoter. A lactose inhibitor LacI binding site was positioned downstream of the TATA box, and the hybrid promoter led to a wide range of dual-mode promoter outputs under the regulation of testosterone and isopropyl β-d-1-thiogalactopyranoside IPTG [147]. In a similar vein, a combination of multiple promoters with different functions can form a sophisticated biosensor system. Quorum sensing was tuned by the pheromone-responsive *FUS1* promoter to sense *α*-pheromone levels. The *ARO9* promoter was responsive to aromatic amino acids in the cultivation medium and was used to drive *α*-pheromone expression, and thus the pheromone quorum sensing could be fine-tuned by the aromatic amino acid concentration [148]. The synergistic effect of multiple regulatory elements or promoters contributed to the multilayer regulation of biosensors.

In conclusion, numerous synthetic promoters have been engineered and their characteristics are shown in Table 2.

## 5. Perspective

Although numerous synthetic promoters have been created to fine-tune gene circuits and metabolic pathways for a greater compatibility and production improvements, the complexity of biological systems still require more sophisticated and elaborate artificial promoters. Although studies have focused on understanding promoter architecture, there remains much to be learned about the interactions of multiple promoter elements to make promoter engineering easier. Promoter designs of the past were unpredictable, requiring laborious screening and testing, which is expensive and time-consuming. In addition, although multiple synthetic promoters with stronger activities were created, they did not exceed the strength of P_GAL1_. Machine learning provides a new design method for synthetic promoters and helps to further understand the mechanism of genome-wide gene expression regulation. Machine learning models may accurately predict synthetic promoter activities and quickly lead to target characteristics. As such, machine learning will likely become a powerful tool for promoter engineering and synthetic biology as a whole.

## Figures and Tables

**Table 1:** Common UASs and URSs in *S. cerevisiae*.

UAS Sequence (5′-3′)	Transcription Factor	Promoters	Inducer	Function	Reference
CGGRNNRCYNYNCNCCG	Gal4p	*GAL1/2/7/10*, *MEL1*	Galactose	Regulation of galactose metabolism	[38]
ATGACTCTT	Gcn4p	*ARG1*, *ARG4*, *HIS4*, *CPA2*	Amino acid starvation	Regulation of amino acid biosynthetic genes	[45]
TTACTAA	Yap1p/2p	*GSH1*, *TRX2*, *YCF1*, *GLR1*	Oxidative stress such as H_2_O_2_	Regulation of genes expressed in response to environmental changes	[46]
TG(A/C)GCCNC	Crz1p	*PMC1*, *PMR1*, *FKS2*	Calcium	Calcineurin-responsive transcription factor	[47,48]
CGGNBNVMHGGA	Cat8p	*FBP1*, *PCK1*, *ACR1*, *IDP2*, *JEN1*	Non-fermentative growth conditions	Derepression of gene expression under non-fermentative growth conditions	[43]
PyPuCACCCPu	Aft1p	*FRE1*, *FTR1*, *FTH1*	Iron deprivation	Iron utilization and homeostasis	[49]
TGAAAC	Ste12p	*TEC1*, *FLO11*	Pheromone	Involved in mating and invasive growth	[50]
GAATGT	Tec1p	*TEC1*, *FLO11*	*n*/A	Ste12p cofactor	[50,51]
CAGCGTG	Hac1p	*KAR2*, *PDI1*, *EUG1*, *FKB2*	Unfolded/misfolded proteins	Regulates the unfolded protein response	[52]
NGAAN	Hsf1p	*HSP82*, *HSP26*, *HSP104*, *HSP26*,	Heat shock	Trimeric heat shock transcription factor	[53]
SYGGGG	Mig1p	*GAL1*, *HXT2*, *SUC2*, *JEN1*	Glucose	Involved in glucose repression	[54]
TGACGTCA	Aca1p	*GRE2*, *COS8*	*n*/A	Basic leucine zipper (bZIP) transcription factor involved in carbon source utilization	[55]
CGGN_3_TNAN_9-12_CCG	Oaf1p	*CTA1*, *FOX1/2/3*, *FAA2*, *PAS8*, *PAS10*	Oleate	Involved in fatty acid beta-oxidation	[56]
TCCGCGGA	Pdr1/3p	*SNQ2*, *PDR5*	Pleiotropic drug	Pleiotropic drug response	[57,58,59]
GGTGGCAAA	Rpn4p	*RPT2/3/6*	Patulin	Regulation of proteasome genes	[60,61]
DNCRCAAAW	Ndt80p	*SMK1*, *SPR3*	Sporulation	Required for full meiotic recombination and middle sporulation	[62]
CCAAT	Hap4p	*CYC1*	Heme	Global regulator of respiratory gene expression	[63]
TGACGTCA	Sko1p	*SUC2*, *MSN2*, *ROX1*, *PTP3*	Osmotic stress	Involved in osmotic and oxidative stress responses	[40,64]
GcCTCGA(G/A)G(C/A)g(a/g)	Xbp1p	*CLN1*, *CYS3*, *SMF2*	Stress or starvation	Transcriptional repressor	[65]
CAC(A/G)T(T/G)	Pho4p	*HIS4*, *PHO5*	Phosphate limitation	Regulation of the purine and histidine biosynthesis pathways	[66]
ACCYYNAAGGT	Zap1p	*ZRT1*, *ZRT2*	Zinc	Zinc-regulated transcription factor	[67]
ACTACTA(T/A)_4_TAG	Smp1p	*STL1*, *CWP1*	Osmotic stress	Osmotic stress response	[68]
CTA(T/A)_4_TAG	Rlm1p	*HKR1*, *KTR2*, *HSP150*, *FLO1*	*n*/A	Maintenance of cell integrity	[69]
TTGGRG	Adr1p	*ADH2*, *ALD4*, *ALD6*, *POX1*	*n*/A	Carbon source responsive transcription factor	[70]
AATCA-N_8_-TGAYT	Vhr1p	*VHT1*, *BIO5*	Biotin	Response to low biotin concentrations	[71]
AAACTGTGG	Met31p	*MET25*, *MET14*, *MET3*	*n*/A	Sulfur amino acid metabolism	[72]
CCCCT	Msn2/4p	*CTT1*, *DDR2*, *HSP12*	Various stress	Response to multiple stress conditions	[73]
CCRTYCRTCCG	Sip4p	*FBP1*, *PKC1*, *ICL1*	*n*/A	Positive regulation of gluconeogenesis	[74]
CGGANNA	Rgt1p	*HXT2*, *HXT4*	Glucose	Glucose-responsive transcription factor	[75,76]
CTTCC	Gcr1p	*ENO1*, *TPI1*, *TDH3*	*n*/A	Transcriptional activator involved in the regulation of glycolysis	[77]
RRRTAACAAGAG	Rox1p	*HEM13*, *COX5B*, *ANB1*, CYC7	Heme	Heme-dependent repressor of hypoxic genes	[78]

Note: *n*/A, not available.

**Table 2:** The characteristics of engineered promoters.

Application	Note (Elements or Parts)	Approach	Expression Range (fold)	Product/Inducers	Reference
Expanding dynamic ranges	P_TEF1_	Random mutation	0–2.0	*n*/A	[15]
	P_TEF1_	Random mutation	0.08–1.2	Increase glycerol 3-phosphate dehydrogenase activity	[127]
	P_ENO2_; P_PDC1_	Random mutation	24.4; 3.0	Obtain a higher cellobiose consumption rate (6.41-fold) and ethanol productivity (6.36-fold)	[128]
	UAS_CLB(3X)_-P_TDH3_; UAS_GAL1_-P_LEUM_/P_CYC_/P_GAL1_	Hybrid	2.5; 50-fold dynamic range	*n*/A	[16]
	UAS_ENO2(3X)_-P_TEF1_; UAS_HXK2_-P_TEF1_/UAS_HSP30_-P_TEF1_	Hybrid	2.0; 8-fold induction range	UAS_HXK2_-P_TEF1_ and UAS_HSP30_-P_TEF1_ are post-diauxic phase-induced promoters	[129]
	P_HIS3_	Manipulating poly(dA:dT) tracts	3-fold dynamic range	*n*/A	[113]
	P_CYC1_	Tuning of nucleosome architecture	6.0	*n*/A	[17]
	PTDH3	Machine learning	1.37	*n*/A	[124]
Reducing homologous recombination	Galactose-inducible promoters	Heterologous expression	2.5-fold to 99-fold induction ratio	Producing 11.5 mg/L lycopene	[130]
	*Psynth* promoters	*De novo*	20-fold dynamic range	*n*/A	[17]
Minimal promoters	UAS_A_/UAS_C_/UAS_FEC_, P_TEF1_	Truncation and hybrid	20-fold dynamic range	*n*/A	[132]
	UAS_EXP1_/UAS_GPD_, P_N30_	Saturation mutagenesis and hybrid	*n*/A	5.5-fold enhancement of lycopene–carotene transformation; producing *β*-carotene 7.4 mg/g DCW	[133]
	UAS_N10_; P_N30_	*De novo* by saturation mutagenesis	0.7	achieve 70% of the strength of the strongest *TDH3* promoter	[135]
Multi-host suitable	P_min_	Random mutation and hybrid	*n*/A	P_bs_ was much stronger than E. coli P_J23119_; 75% of that of P_cdd_ in B. subtilis; lower than that of the strong promoter P_TDH3_	[137]
	CRM; P_AOX1_	Computational design and hybrid	200-fold dynamic range	0.3% to 70.6% of the wild type P_AOX1_ level	[138]
Biosensor	P_TRX2_	Hybrid	100-fold dynamic range	NADPH/NADP+ ratio	[139]
	P_YGP1_; P_CCW14_	Hybrid	6.0; 16.0	Enabling a 10-fold increased production of lactic acid; low pH	[140]
	P_CYC1_	Hybrid	1000-fold induction ratio	Tetracycline	[142]
	P_CYC1_	Hybrid	*n*/A	Camphor	[141]
	P_GAL1_	Hybrid	*n*/A	Fatty acid/fatty acyl-CoA	[145]
	P_GPM1_	Hybrid	1-fold to 4.17-fold induction ratio	Enhancing 3-hydroxypropionic acid titer by 120%; Malonyl-CoA biosensor	[146]
	P_CYC1_	Hybrid	8-fold induction ratio	IPTG and testosterone dual induction	[147]

Note: *n*/A, not available.
